# Electrochemical Molecular Conversion of α-Keto Acid to Amino Acid at a Low Overpotential Using a Nanoporous Gold Catalyst

**DOI:** 10.3390/ijms22179442

**Published:** 2021-08-31

**Authors:** Yasuhiro Mie, Shizuka Katagai, Chitose Mikami

**Affiliations:** Bioproduction Research Institute, National Institute of Advanced Industrial Science and Technology (AIST), 2-17-2-1, Tsukisamu-higashi, Toyohira, Sapporo 062-8517, Japan; s.katagai@aist.go.jp (S.K.); mikami-c@aist.go.jp (C.M.)

**Keywords:** biomass-derivable, amino acid, electrosynthesis, nanoporous gold, anodization

## Abstract

A nanoporous gold (NPG) electrode prepared through a facile anodization technique was employed in the electrochemical reductive amination of biomass-derivable α-keto acids in the presence of a nitrogen source to produce the corresponding amino acids. NPG showed a clear reductive current in the presence of α-keto acid and NH_2_OH, and the electrolysis experiments confirmed the production of L-amino acid. A reductive voltammetric signal at the NPG electrode appeared at a more positive potential by 0.18–0.79 V, compared with those at the planar-gold electrode without anodization and other previously reported electrode systems, indicating the high activity of the prepared nanostructure for the electrochemical reaction. Maximum Faradaic efficiencies (FEs) of 74–93% in the reductive molecular conversion to amino acids of Ala, Asp, Glu, Gly, and Leu were obtained under the optimized conditions. The FE values were strongly dependent on the applied potential in the electrolysis, suggesting that the hydrogen evolution reaction at the electrode surface was more significant as the applied potential became more negative. The effect of potential at the NPG was lower than that at the planar-gold electrode. These results indicate that nanostructurization decreases the overpotential for the electrochemical reductive amination, resulting in high FE.

## 1. Introduction

As the demand for a sustainable society increases, efforts have been made to develop environmentally friendly methods for chemical production [1]. The electrochemical molecular conversion of redox-active species is one of the useful strategies to synthesize organic molecules in addition to sensor and energy conversion technologies as it can be conducted with electricity from renewable energy sources [2,3,4]. Furthermore, as the electrochemical system can be easily miniaturized, it has advantages, especially for the distributed on-site production of high-value chemicals in on-demand needs [1].

In electrochemical methods, an electrode works as a catalyst and an electron server/receiver. Therefore, in addition to the nature of the electrode materials, its surface structure is a key factor that determines the catalytic activity. Owing to the growth of nanotechnological methods, electrodes composed of nanomaterials and the nanostructurization of electrode surfaces have been developed [5,6,7]. Now, it is well-recognized that the nanoarchitecture of electrodes significantly enhances the electrocatalytic activity, and one of the reasons is the abundant low-coordinated surface atoms at the steps/kinks [8,9,10]. Among many types of nanostructured electrodes, nanoporous metals have been attracted in many fields owing to their large surface area [11,12]. Nanoporous gold (NPG) is promising in electrochemical applications of catalysis and sensing, owing to its advantages, such as high conductivity, catalytic activity, chemical inertness, physical stability, reusability, and facile surface modification. Several efficient electrocatalytic reactions using NPG, including the reduction of CO_2_ and H_2_O_2_, oxidation of alcohol, glucose, CO, and aromatic compounds, and hydrogenation reactions of alkynes and alkenes, have been reported [13,14,15,16,17,18,19,20]. To date, NPG has been mostly prepared by the selective etching of a less noble component in gold-containing metal alloys. Its catalytic properties for redox-active molecules have been extensively studied, and the enhanced activity has been discussed [21,22,23]. Recently, a more facile and time-saving method, where NPG is fabricated by simply anodizing conventional planar (commercially available) gold electrodes, has been reported [24] and developed [25,26,27,28,29]. Anodization in a solution containing a high (0.5–2 M) Cl^−^ concentration is a rapid (within 2 min) strategy for producing NPG composed of ligaments and pores [24,25], which are similar to those prepared using the conventional dealloying method described above. NPG with thinner ligaments was also prepared by anodization in a solution of lower Cl^−^ concentrations [28,29]. An enhanced electrocatalytic activity of anodized NPGs was reported for such reactions as O_2_ reduction, CO_2_ reduction, and monosaccharide oxidation [30,31,32], revealing the potency of NPGs.

Currently, α-keto acid derivatives can be produced from lignocellulosic biomass, which can be a non-food-competing chemical feedstock [33,34,35]. Hence, the electrochemical reductive amination of keto acids to produce the corresponding amino acids is desired as an environmentally friendly method as amino acids are useful materials in the industry, and the current production system with a microbial fermentation process or chemical synthesis has several challenges, such as low efficiency for some amino acids, high energy consumption, and the use of hazardous chemicals [33,36,37,38]. Unfortunately, studies on the electrochemical conversion of α-keto acids to amino acids are very limited, and previous reports employed Hg, Pd, Pt, and graphite electrodes [39,40,41]. Recently, Fukushima et al. reported the electrochemical production of seven amino acids from the corresponding α-keto acids using the anatase-TiO_2_-based electrode with an extremely high Faradaic efficiency (FE) of ~99% at 0 °C [33]. However, gold materials have not been used to investigate the aforementioned electrochemical conversion, and the effect of nanostructures on the reaction is unknown.

**Figure 1:**
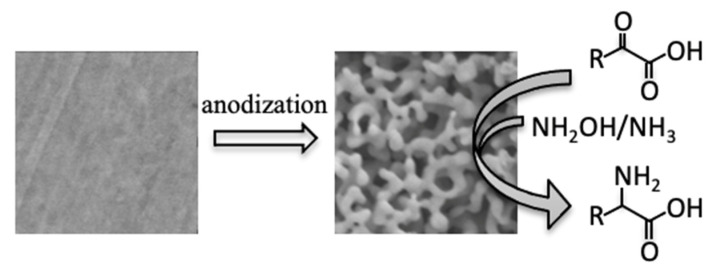
Schematic representation of electrochemical molecular conversion: nanostructurization for a commercially available planar-gold electrode surface and the electrochemical reductive amination of α-keto acid in the presence of a nitrogen source to produce L-amino acid.

In this study, the nanostructurization of gold electrodes significantly improved the electrochemical reductive amination of α-keto acids to produce the corresponding L-amino acids (Figure 1). We demonstrated that NPG enables the amino acid production at a lower overpotential, which separates the reductive amination reaction from the competing hydrogen evolution reaction (HER) at the gold surface, resulting in a high FE of ~93% for the electrochemical molecular conversion.

## 2. Results and Discussion

### 2.1. Electrochemical Reduction of Ketoglutaric Acid at NPG and Conventional (Planar) Gold Electrodes in the Presence of a Nitrogen Source

**Figure 2:**
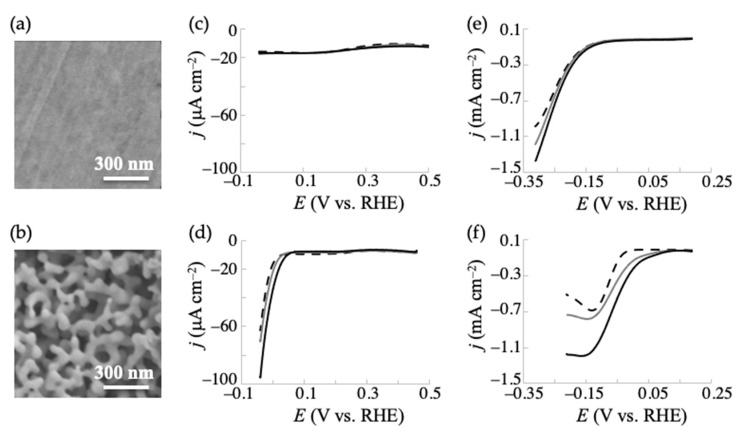
Surface morphologies of (**a**) planar (nonanodized) gold and (**b**) anodized NPG electrodes observed by SEM. (**c**–**f**) Cyclic voltammograms obtained at the electrodes. Voltammograms for 25 mM (gray line) and 50 mM (black line) of α-ketoglutaric acid measured in (**c**,**d**) 1.5 M of NH_3_/NH_4_Cl solution (pH 10) and (**e**,**f**) 0.15 M of NaCl solution containing 50 mM of NH_2_OH (pH 1.6) at (**c**,**e**) planar-gold and (**d**,**f**) NPG electrodes. Dashed lines indicate voltammograms recorded without α-ketoglutaric acid and NH_2_OH.

First, we targeted α-ketoglutaric acid (2-oxoglutaric acid) and conducted voltammetry and electrolysis to investigate whether gold materials enable the electrochemical conversion of the α-keto acid to the corresponding amino acid (L-glutamic acid) in the presence of a nitrogen source. The morphologies of the gold electrodes, i.e., the conventional planar (without anodization) gold and anodized NPG, are shown in Figure 2a,b. The anodization process yielded a nanoporous structure composed of ~50 nm sized ligaments on the material, consistent with previous reports [28,29]. NPG with a roughness factor (*R*_f_) of about 5 (electrode surface area = 0.35 ± 0.10 cm^−2^) was used herein as higher *R*_f_ values result in deeper nanoporous layers in the NPG structure, which hinders the diffusion of the target molecule deep inside of the porous structure [29].

According to the previous studies [33,39,40,41], NH_3_ and NH_2_OH were used as nitrogen sources in the electrochemical reductive amination at gold electrodes. The former and the latter were examined in 1.5 M of NH_3_/NH_4_Cl buffer (pH 10) and in 0.15 M of NaCl solution containing 50 mM of NH_2_OH (pH was adjusted to 1.6), respectively. The linear sweep voltammograms obtained at the planar-gold and NPG electrodes in the presence and absence of α-ketoglutaric acid at a potential scan rate of 5 mV s^−1^ are shown in Figure 2c–f. When NH_3_ was used as the nitrogen source, no significant reductive current was observed at the planar-gold electrode (Figure 2c), and a relatively small increase in current (compared with the previous study [33] and undermentioned NH_2_OH case) at the NPG electrode starting at around 0.03 V (Figure 2d) upon the addition of α-ketoglutaric acid was observed. The lower activity compared with the below NH_2_OH case agrees with the previous report [33], and the significantly smaller current changes herein indicate that the gold material was not effective for the reductive amination of α-ketoglutaric acid with NH_3_ (pH 10) as the nitrogen source. On the other hand, when NH_2_OH was used, a significant increase in the reductive current was observed in the presence of α-ketoglutaric acid at both planar-gold and NPG electrodes (Figure 2e,f), especially at the latter. The current increased with the concentration of α-ketoglutaric acid, indicating the electrochemical reductive reaction of the substance. Importantly, the NPG surface exhibited the reductive current starting at around 0.1 V, which was a much more positive potential by 0.18–0.30 V compared with those at the planar-gold (Figure 2e) and previously reported TiO_2_ electrodes (with a scan rate of 10 mV s^−1^ and pH 0.53) [33] with NH_2_OH as the nitrogen source. It was also much more positive by ~0.79 V compared with those obtained at Hg, Pt, Pd, and graphite electrodes with NH_3_ as the nitrogen source [39,40,41]. These indicate that the present anodized NPG could be a useful catalyst for the electrochemical production of L-glutamic acid from α-ketoglutaric acid in the presence of NH_2_OH.

**Figure 3:**
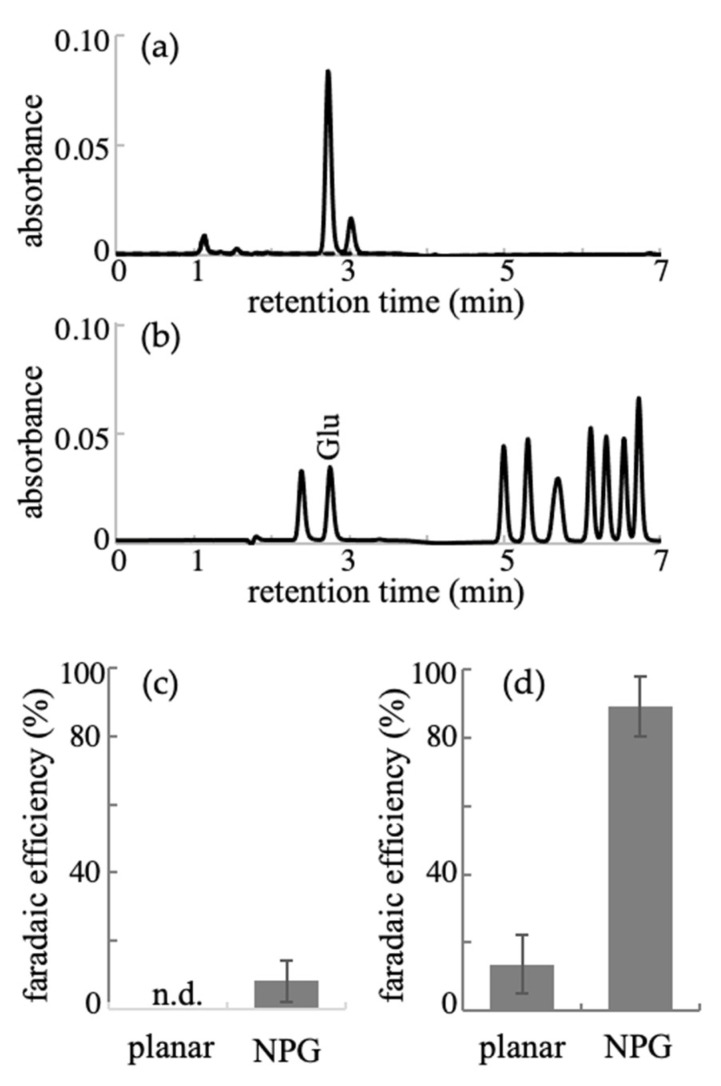
Typical HPLC chromatograms of (**a**) electrolyzed solution for 50 mM of α-ketoglutaric acid in the presence of 50 mM of NH_2_OH using anodized NPG electrode for 30 min and (**b**) standard solutions containing 1 nmol of L-amino acids. The dotted line in (**a**) indicates the chromatogram of the solution before electrolysis. Faradaic efficiency (FE) for electrochemical production of L-glutamic acid at planar-gold and NPG electrodes in the presence of (**c**) NH_3_ and (**d**) NH_2_OH as nitrogen sources. EF values are represented as mean ± SD (*n* = 3 independent experiments). “n.d.” means not detected.

To confirm the formation of L-glutamic acid in the above electrochemical reduction reaction, electrolysis experiments of a 50 mM α-ketoglutaric acid solution were conducted. The electrolysis time was set for 120 min and 30 min for the planar-gold and NPG electrodes, respectively, in consideration of the difference in each electrode surface area. The electrolyzed solutions were analyzed by high-performance liquid chromatography (HPLC) with a phenyl isothiocyanate prelabeling strategy and enzymatic L-glutamic acid assay with a redox dye system of the commercially available kit. Applied potentials of 0.02 and −0.04 V were used for the electrolysis with NH_3_ and 50 mM of α-ketoglutaric acid at the NPG and planar-gold electrodes, respectively, whereas −0.01 and −0.11 V were employed in the case of NH_2_OH at NPG and planar-gold electrodes, respectively. Figure 3a shows the typical chromatogram recorded for the electrolyzed solution of 50 mM of α-ketoglutaric acid and NH_2_OH using NPG for 30 min and at an applied potential of −0.01 V. Compared with the chromatogram of the standard L-amino acid solution (Figure 3b), the formation of L-glutamic acid was evident, indicating successful electrochemical reductive amination [33]. The enzymatic assay also indicated the production of a similar amount of L-glutamic acid (not shown here). To evaluate the efficiency of the present electrochemical conversion, the FE was evaluated using the charge passed through during the electrolysis and the amount of produced L-glutamic acid. Referring to the previous electrochemical study of α-keto acids in the presence of a nitrogen source, we assumed that the conversion of α-keto acids to L-amino acids proceeds with two- and four-electron reduction through imine and oxime as a nitrogenated intermediate in the presence of NH_3_ and NH_2_OH, respectively [33]. Figure 3c,d show the averaged FE values calculated for the reduction in the solution containing NH_3_ and NH_2_OH at both the planar-gold and NPG electrodes. In the case of NH_3_, no L-glutamic acid was produced at the planar-gold electrode, and a very small amount was produced at the NPG electrode, resulting in an FE of 5%. This is reasonably accepted in consideration of the voltammetric responses described above. Again, a lower activity of the gold electrodes for the electrochemical reductive amination of α-keto acid in NH_3_/NH_4_Cl solution was observed. On the other hand, as for the NH_2_OH case, FE values of 16% and 89% were obtained at the planar-gold and NPG electrodes, respectively. The efficiency at the NPG electrode was much higher than that at the planar-gold, and the catalytic activity of the gold electrode was significantly improved by nanostructurization (facile anodization treatment). Combined with the voltammetric results, where the reductive conversion was observed with a lower overpotential at the NPG electrode, we conclude that the NPG surface is an effective catalyst for converting biomass-derivable α-keto acid with NH_2_OH as a nitrogen source to the corresponding L-amino acid with a high FE and low overpotential. The electrochemical sensing applications of NPG, particularly that prepared using a dealloying method, have been studied extensively [42,43,44]. The results showed that the nanoporous structure enhances the electrochemical signals and lowers the overpotential for redox reactions. The present anodized NPG has a similar shape to that of the dealloyed NPG. Therefore, the higher current at lower overpotential in the present α-keto acid reduction agrees with the aforementioned reports.

### 2.2. Effect of Applied Potential on the Electrochemical Production of L-Glutamic Acid

**Figure 4:**
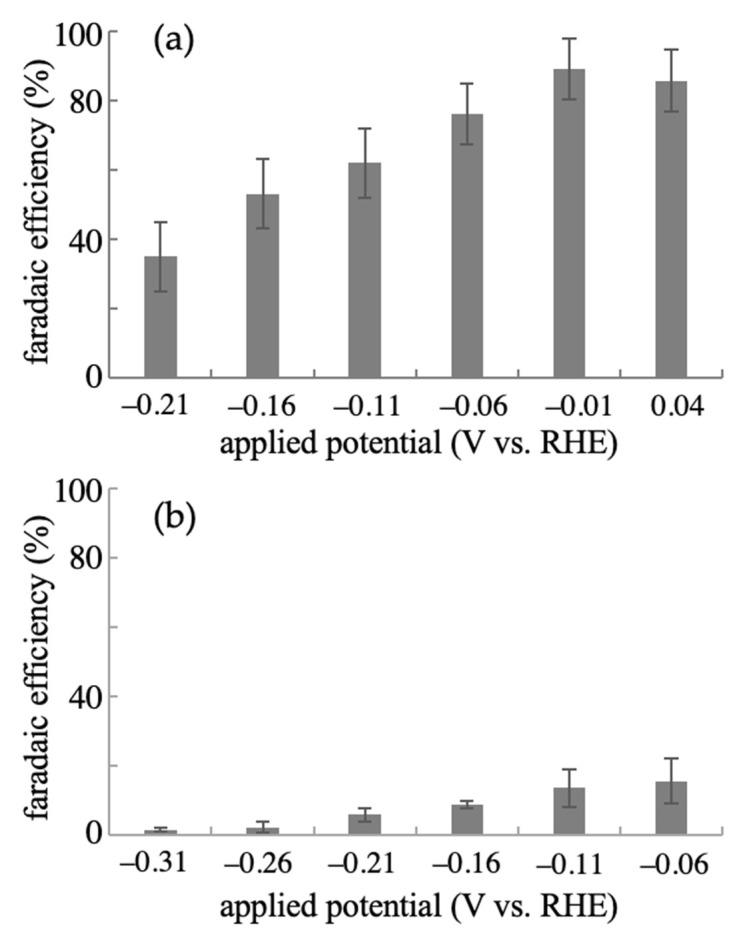
Relationship between FE of L-glutamic acid production and applied potentials for the electrolytic reaction of 50 mM of α-ketoglutaric acid in the presence of 50 mM of NH_2_OH using (**a**) NPG and (**b**) planar-gold electrodes. FE values are presented as mean ± SD (*n* = 3 independent experiments).

The effective catalytic properties of the anodized NPG for the electrochemical reductive amination of α-ketoglutaric acid using NH_2_OH as a nitrogen source were demonstrated as described above. The applied potential in the electrochemical molecular conversion is one of the key factors affecting reaction efficiency. We examined the effect of applied potential on the electrolysis of 50 mM of α-ketoglutaric acid with an equivalent amount of NH_2_OH using both planar-gold and NPG electrodes for 120 min and 30 min, respectively. The optimized concentration of α-ketoglutaric acid of 50 mM (Figure S1) was selected. From the linear sweep voltammograms (Figure 2e,f), onset potentials of approximately −0.06 and 0.12 V for the reductive current for α-ketoglutaric acid were estimated at the planar-gold and NPG electrodes, respectively. Hence, applied potentials more negative than these values for the electrolysis were set. As shown in Figure 4, the FE for the electrochemical production of L-glutamic acid from α-ketoglutaric acid was strongly dependent on the applied potential in the electrolysis at both the planar-gold and NPG electrodes. For the NPG electrode, as the applied potentials became negative, the FE gradually decreased. The FE for the production of L-glutamic acid obtained at −0.21 V was about 40% of that observed at 0.04 V. A similar tendency was observed at the planar-gold electrode, and the effect of potential was higher than that at the NPG electrode. At −0.310 V, the FE was less than 10% compared with that at −0.06 V. The NPG electrode exhibited the highest averaged FE of ~90% at potentials more positive than −0.01 V, which is close to the highest value (96.7%) in a previous study at a TiO_2_-based electrode with an applied potential of −0.5 V. On the other hand, the maximum value obtained at the planar-gold electrode was 16% at a potential of −0.06 V. These results indicate that the anodized nanostructure considerably improves the electrocatalytic activity for the conversion of α-ketoglutaric acid to the glutamic acid in the presence of a nitrogen source and can be an effective catalyst for the reaction.

Considering the pH of 1.6 used for the electrochemical conversion, the HER should be considered at gold material electrodes, especially for the nanostructured ones [42,43,44]. HERs on metallic-based electrodes compete with and inhibit the target/desired reactions, such as the electrochemical reduction of water-soluble carboxylic acids [45]. As shown in Figure 2e,f (dotted lines), the planar-gold and NPG electrodes exhibited a significant reductive current at potentials more negative than −0.11 and −0.01 V, respectively, in the absence of NH_2_OH and α-ketoglutaric acid, indicating the occurrence of HER, which decreases the FE of the electrosynthesis of L-glutamic acid under these potentials. The reductive current signal of HER (dashed line in Figure 2e,f) against the total reductive signal (solid line in Figure 2e,f) at the planar-gold electrode was larger than that at the NPG electrode. These voltammetric features are consistent with the effect of the applied potential on the FE in L-glutamic acid production. These results suggest that nanostructurization of the surface of the planar-gold electrode by the facile anodization process improves the activity for the reduction of α-keto glutaric acid than for HER. Hence, the two electrochemical reductive reactions could be separated in the electrolysis experiments at appropriate applied potentials with the NPG electrode, resulting in a high FE. A previous study reported that a conventional planar gold and NPG electrode have different compositions of surface crystallographic orientations [29]. Therefore, the orientation of NPG could favor an interaction with the reactant/intermediate for the reduction of α-keto acids compared to that of the HER. The details of this phenomenon are under investigation.

### 2.3. Reusability and Generality 

**Figure 5:**
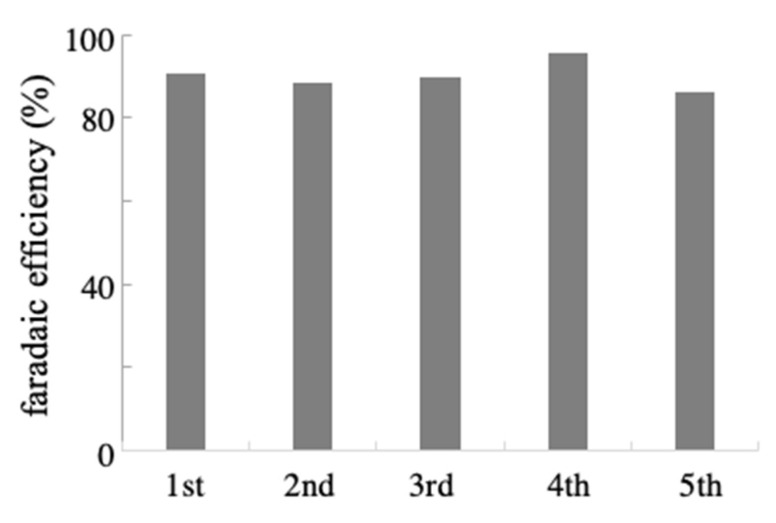
FE for L-glutamic acid production obtained at a single NPG electrode from five sequential experiments. Electrolysis was conducted on 50 mM of α-ketoglutaric acid in the presence of NH_2_OH at −0.01 V for 30 min.

To further investigate the efficacy of the NPG electrode for the electrochemical synthesis of amino acids from α-keto acids, reusability tests for the electrode were conducted. Figure 5 shows the FE values evaluated from five sequential reductive electrolysis reactions for α-ketoglutaric acid in the presence of the corresponding amount of NH_2_OH under a fixed condition using a single NPG electrode. The NPG electrode showed almost constant FE values, indicating less deterioration of the activity of the electrode surface during the experiments. This was also supported by the similar surface morphology of NPG before and after the electrolysis experiments (Figure S2).

**Figure 6:**
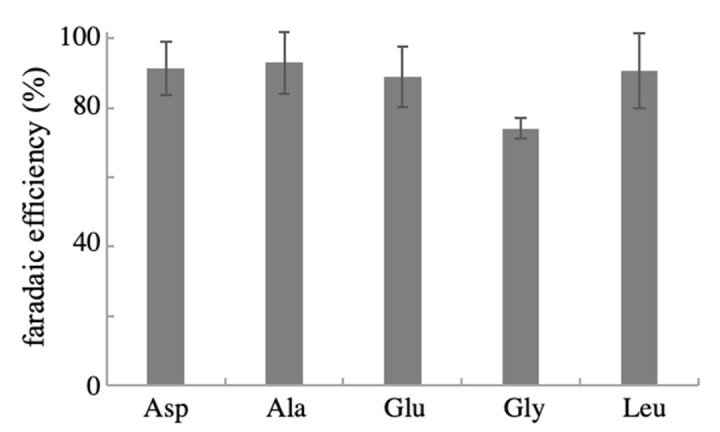
FE for the electrochemical conversion of α-keto acids with NH_2_OH to the corresponding amino acids using an NPG electrode. A solution containing 50 mM of α-keto acid and NH_2_OH was electrolyzed at −0.01 V for 30 min. The FE values are presented as mean ± SD (n = 4 independent experiments).

Finally, we examined if the NPG can be used for the conversion of other α-keto acids to produce the corresponding L-amino acids. Fukushima et al. [33] reported that alanine, aspartic acid, glutamic acid, glycine, and leucine were electrosynthesized with a high FE of 91–99% from the corresponding α-keto acids, i.e., pyruvic, oxaloacetic, α-ketoglutaric, glyoxylic, and 4-methyl-2-oxovaleric acid, respectively, using an anatase TiO_2_ electrode with an applied potential of −0.5 V. Herein, the α-keto acids were converted in the presence of NH_2_OH using the anodized NPG electrode with an applied potential of −0.01 V, which was sufficient to reduce all the α-keto acids tested in this study. HPLC analysis and an enzymatic assay of each electrolyzed solution revealed the successful production of the corresponding L-amino acids. The calculated FEs are depicted in Figure 6. All amino acids tested were produced with an FE of 74–93%. Except for Gly, whose average FE was significantly small (74%) (the reason is yet to be clarified), the FE for the production of other amino acids (89–93%) was close to the highest values in previous reports [33]. Hence, the anodized NPG electrode has high potency for the electrochemical production of L-amino acids from the biomass-derivable α-keto acids, and the nanostructurization of gold materials would be effective for developing catalytic electrodes for electrochemical molecular conversions.

## 3. Materials and Methods

### 3.1. Materials 

Oxaloacetic acid, α-ketoglutaric (2-oxoglutaric) acid, ammonia solution, hydroxyammonium sulfate, amino acids standard solution (Type H), phenyl isothiocyanate (PITC), PTC-amino acid mobile phase solutions A and B, and a Wakosil-PTC column were purchased from FUJIFILM Wako Pure Chemical Corporation (Osaka, Japan). Pyruvic and glyoxylic acids were purchased from Tokyo Chemical Industry Co., Ltd. (Tokyo, Japan). 4-Metyl-2-oxovaleric acid and an L-amino acid quantification kit were purchased from Sigma-Aldrich Co. LLC (St. Louis, MO, USA). An L-glutamate kit YAMASA NEO was purchased from Yamasa Corporation (Choshi, Japan). Electrodes, including gold disks (ø = 3 mm), Pt coils, and Ag|AgCl|sat. KCl, were purchased from BAS Inc., (Tokyo, Japan).

### 3.2. Electrode Preparation

A gold disk electrode was polished with 1 and 0.125 µm of diamond slurries, followed by 0.05 µm of alumina slurry, and electrochemically cleaned in a 0.5 M H_2_SO_4_ solution. To prepare the NPG electrode, first, the cleaned gold disk was immersed in a 0.5 M HCl solution, and linear sweep voltammetry was conducted to evaluate the passivation potential, where the gold electrode surface was passivated by the formation of an oxide layer with a sudden current drop after the flow of the gold dissolution current [24,25,29]. Then, the constant potential for nanostructurization (anodization), which was set to be more negative by 5–30 mV from the passivation potential, was applied for 0.5–7 min. The morphology of the anodized surface of the electrode was analyzed by scanning electron microscopy (SEM) using an S-4300 FE-SEM (Hitachi Ltd., Tokyo, Japan). The actual electrode surface area was determined from the reductive charge of gold oxides in the cyclic voltammograms measured in a 0.5 M H_2_SO_4_ solution [46,47,48,49]. The roughness factor (*R*_f_) of prepared NPG was calculated by dividing the electrode surface area by the geometrical surface area of the gold disk electrode. The *R*_f_ values of NPG electrodes were arranged by controlling the time for the anodization process.

### 3.3. Electrochemical Measurements 

All electrochemical measurements were conducted using an ALS model 660D electrochemical analyzer (CH Instruments Inc., Austin, TX, USA). A normal three-electrode configuration consisting of an Ag/AgC|sat. KCl reference electrode, a Pt auxiliary electrode, and a cleaned commercially available conventional planar-gold or NPG working electrode was employed. Potentials in the results were converted to the reversible hydrogen electrode (RHE) reference [33]. Voltammetry was performed for the potential ranges of −0.3 to 0.5 V, depending on the experimental conditions at potential scan rates of 5–50 mV s^−1^. 

### 3.4. Electrolysis and Product Analysis 

The α-keto acids were electrolyzed using the aforementioned three-electrode configuration for 30 and 120 min at the NPG and conventional planar-gold electrodes, respectively, under a constant applied potential. The Pt auxiliary electrode was separated by ion-permeable porous glass from the working electrode. The electrolyzed solutions, which were expected to have the corresponding L-amino acids, were analyzed by HPLC (Chromaster, Hitachi High-Teck Corp., Tokyo, Japan). The reaction solutions were dried and treated with a PITC solution (the L-amino acids were modified by PITC) according to the manufacturer’s instructions. The prelabeled reactants were applied to the Wakosil-PTC column and eluted with a linear gradient from 0% to 70% of the aforementioned mobile phase B. The L-amino acids in the electrolyzed solutions were also determined enzymatically with the commercially available colorimetric assay kits following the manufacturers’ instructions.

## 4. Conclusions

The anodized NPG surface was employed for the electrochemical molecular conversion of biomass-derivable α-keto acids in the presence of a nitrogen source to the corresponding L-amino acids. The surface nanostructurization enabled the reductive amination reaction at a lower overpotential and significantly improved the conversion efficiency. As the applied potential for the electrochemical conversion became more positive, the L-amino acid production efficiency increased. FE values of 89–93% were obtained for the production of Ala, Asp, Glu, and Leu at the NPG electrode. These were much higher than that (16% at maximum) at the conventional planar-gold electrode (without nanostructurization) and close to the highest values (~99%) reported for TiO_2_ electrodes. These results demonstrate that the present facile nanostructurization is effective for improving the reductive amination of α-keto acids. They also indicate that it would be useful to develop an electrode catalyst for the electrochemical molecular conversions of redox active species.

## Data Availability

The data presented in this study are available on request from the corresponding author.

## Supplementary Materials

The following are available online at https://www.mdpi.com/article/10.3390/ijms22179442/s1, Figure S1: FE for L-glutamic acid production obtained at the NPG electrode as a function of the a-ketoglutaric acid concentration; Figure S2: Scanning electron microscopy images of the NPG electrode before and after the electrolysis experiments.
