# Supplementary material for: Electrochemical Molecular Conversion of α-Keto Acid to Amino Acid at a Low Overpotential Using a Nanoporous Gold Catalyst
Source: Int J Mol Sci. 2021 Aug 31;22(17):9442. doi: 10.3390/ijms22179442 (PMC8431653; doi:10.3390/ijms22179442)
Supplement: Supplementary file 1 [file ijms-22-09442-s001.zip › ijms-1343229-supplementary.pdf]

– Supplementary Figures –

Electrochemical Molecular Conversion of α-Keto Acid to Amino Acid at a Low Overpotential Using a Nanoporous Gold Catalyst

Yasuhiro Mie *, Shizuka Katagai and Chitose Mikami

Bioproduction Research Institute, National Institute of Advanced Industrial Science and Technology (AIST), 2-17-2-1, Tsukisamu-higashi, Toyohira, Sapporo 062-8517, Japan; s.katagai@aist.go.jp (S.K.); mikami-c@aist.go.jp (C.M.)

** Corresponding Author: Yasuhiro Mie*

Tel.: +81-11-857-8913; Fax: +81-11-857-8915;
Email: yasuhiro.mie@aist.go.jp

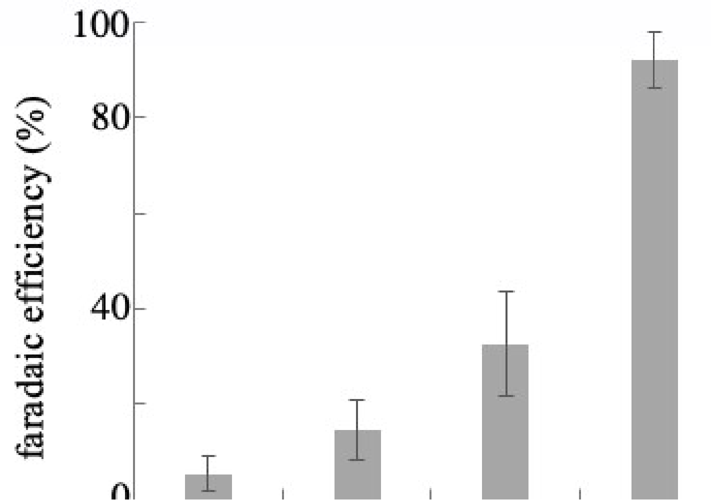

Figure. S1. FE for L-glutamic acid production obtained at the NPG electrode as a function of the α-ketoglutaric acid concentration. Electrolysis was conducted in the presence of NH_2OH for 30 min.

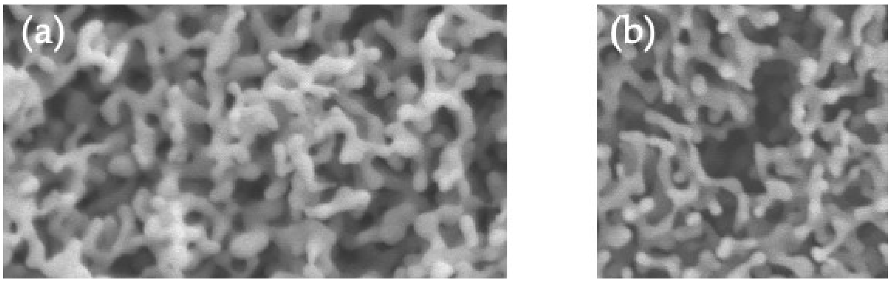

Figure. S2. Scanning electron microscopy images of the NPG electrode before (a) and after (b) the electrolysis experiments under the conditions described in Figure 5 in a 1.5 × 1.0 μm region.
